# Retroperitoneoscopic ureterolithotomy to treat large ureteral stones in the proximal ureter

**DOI:** 10.1590/S1677-5538.IBJU.2019.0272

**Published:** 2020-09-02

**Authors:** Victor Srougi, Fabio C. Torricelli, Hiury S. Andrade, Marco A. Arap, Anuar I. Mitre, Eduardo Mazzucchi, Ricardo J. Duarte, Miguel Srougi

**Affiliations:** 1 Hospital das Clínicas Universidade de São Paulo Faculdade de Medicina SP Brasil Divisão de Urologia, Hospital das Clínicas, Universidade de São Paulo, Faculdade de Medicina, SP, Brasil

## Abstract

**Introduction:**

Retroperitoneoscopic ureterolithotomy emerged as an option for the extraction of large stones in the proximal ureter, offering short convalescence and low rates of residual fragments.

**Materials and methods:**

We describe the case of a 50-year-old male, who presented at our emergency department with right flank pain for 15 days without fever. He had a past medical history of nephrolithiasis. A non-contrast computed tomography (NCCT) evidenced a stone with 1.5cm and 1200HU in the right proximal ureter associated with ipsilateral hydronephrosis. A retroperitoneoscopic ureterolithotomy was planned.

**Results:**

The surgery was performed under general anesthesia, with the patient in 90o left lateral decubitus. Retroperitoneal space was created with blunt finger dissection. Three ports were used and the operative time was 60 minutes. Foley catheter was removed the morning after the procedure and the drain 8 hours later. The patient was discharged in the first postoperative day. Double J catheter withdrawal was done 4 weeks after surgery. No intraoperative or postoperative (90-days) complications were recorded. Control NCCT demonstrated the complete removal of the ureteral stone.

**Conclusion:**

The retroperitoneoscopic approach is an effective alternative to treat large proximal ureteral stones.

